# Characterising sex‐related differences in lower‐ and higher‐threshold motor unit behaviour through high‐density surface electromyography

**DOI:** 10.1113/EP091823

**Published:** 2024-06-18

**Authors:** Edoardo Lecce, Alessandra Conti, Stefano Nuccio, Francesco Felici, Ilenia Bazzucchi

**Affiliations:** ^1^ Department of Movement, Human and Health Sciences, Laboratory of Exercise Physiology University of Rome ‘Foro Italico’ Rome Italy

**Keywords:** central nervous system, electromyogram, HDsEMG, motor control, motor unit, skeletal muscle

## Abstract

**Abstract:**

Emerging questions in neuromuscular physiology revolve around whether males and females share similar neural control in diverse tasks across a broad range of intensities. In order to explore these features, high‐density electromyography was used to record the myoelectrical activity of biceps brachii during trapezoidal isometric contractions at 35% and 70% of maximal voluntary force (MVF) on 11 male and 13 female participants. Identified motor units were then classified as lower‐threshold (recruited at ≤30%MVF) and higher‐threshold (recruited at >30%MVF). The discharge rate, interspike interval variability, recruitment and derecruitment thresholds, and estimates of neural drive to motor neurons were assessed. Female lower‐threshold motor units showed higher neural drive (*P *< 0.001), accompanied by higher discharge rate at recruitment (*P *= 0.006), plateau (*P *= 0.001) and derecruitment (*P *= 0.001). On the other hand, male higher‐threshold motor units showed greater neural drive (*P = *0.04), accompanied by higher discharge rate at recruitment (*P *= 0.005), plateau (*P *= 0.04) and derecruitment (*P *= 0.01). Motor unit discharge rate normalised by the recruitment threshold was significantly higher in female lower‐threshold motor units (*P *< 0.001), while no differences were observed in higher‐threshold motor units. Recruitment and derecruitment thresholds are higher in males across all intensities (*P *< 0.01). However, males and females have similar activation and deactivation strategies, as evidenced by similar recruitment‐to‐derecruitment ratios (*P *> 0.05). This study encompasses a broad intensity range to analyse motor unit sex‐related differences, highlighting higher neural drive and discharge rates in female lower‐threshold motor units, elevated recruitment and derecruitment thresholds in males, and convergences in activation and deactivation strategies.

**Highlights:**

- **What is the central question of the study?**
  Do male and female motor units behave similarly in low‐ and high‐intensity contractions?
- **What is the main finding and its importance?**
  Female motor units show higher discharge rates in low‐intensity tasks and lower discharge rates in high‐intensity tasks, with no differences in recruitment behaviour. A broader inter‐spike interval variability was also observed in females. These findings underline that there are sex‐specific differences concern the firing strategies based on task intensity.

## 1 INTRODUCTION

Recent evidence has shed light on neuromuscular system complexity by studying motor units (MUs), representing the functional units and the actual link between the neural and the musculoskeletal systems (Del Vecchio, Negro, et al., 2019; Germer et al., 2021; Holobar et al., 2009; Nuccio et al., 2021). However, a dimension of their complexity that has gained prominence only recently relates to sex‐dependent differences (Guo, Jones, et al., 2022; Handelsman et al., 2018; Harrison et al., 2023; Inglis & Gabriel, 2020; Jenz et al., 2023; Lulic‐Kuryllo & Inglis, 2022; Olmos et al., 2023).

The recognition of distinct motor control strategies in individuals of different sexes has brought forth a new era of inquiry in the fields of physiology, kinesiology and sports science (Jo et al., 2022; Mcdougall et al., 2023; Piasecki et al., 2021; Taylor et al., 2022). While numerous studies have documented disparities in skeletal muscle mass, strength and endurance capacities between males and females, the underlying neuromuscular mechanisms governing these differences remain a subject of active investigation (Lulic‐Kuryllo & Inglis, 2022; Olmos et al., 2023). Previous evidence highlighted differences in surface electromyography (sEMG) parameters, reporting greater root mean square values in male participants (Pincivero et al., 2000) accompanied by a lower firing variability (Ahamed et al., 2015). To understand the physiological mechanisms explaining these distinctions, researchers have redirected their attention towards investigating the inherent differences in motor unit properties (Guo, Jones, et al., 2022; Jenz et al., 2023; Olmos et al., 2023; Piasecki et al., 2021; Taylor et al., 2022), including recruitment thresholds, firing rates and contractile characteristics, between males and females. Nevertheless, a definitive determination of whether reported disparities can be attributed to motoneuronal and musculoskeletal factors rather than variations in hormonal fluctuations remains elusive (Handelsman et al., 2018; Lulic‐Kuryllo & Inglis, 2022; Piasecki et al., 2023, 2024; Smith & Woolley, 2004; Velders & Diel, 2013; Wierman, 2007).

Higher motor unit discharge rate in females than males has been reported at 10% and 25% maximal voluntary force (MVF) using intramuscular‐EMG on vastus lateralis (VL), along with no differences in force steadiness (Guo, Jones, et al., 2022). Moreover, recent findings obtained through high‐density surface electromyography (HDsEMG) revealed similar discharge rates in 30%MVF ramp contractions (recruitment+derecruitment, no steady phase) with broader persistent inward current in the lower limb (tibialis anterior (TA), gastrocnemius (GM), soleus (SOL)) muscles (Jenz et al., 2023). However, a higher firing rate in the first dorsal interosseus (FDI) was found at 10%, 30% and 60%MVF (Nishikawa et al., 2024) and in 20%MVF ramp (recruitment+steady phase+derecruitment) contractions in TA (Taylor et al., 2022). Furthermore, previous findings (TA, intramuscular EMG) reported significant differences at high‐intensity contractions (Inglis & Gabriel, 2020), underlining higher discharge rate in females in tasks reaching up to 60%MVF and matching values within the 60−80%MVF range, whereas motor unit signal amplitude has been demonstrated (Parra et al., 2020) to be wider in males at higher intensities (70%MVF, first‐dorsal interosseus, sEMG).

Since previous findings mainly concern overall myoelectrical activity (sEMG) and motor unit activity in low‐intensity tasks (HDsEMG) in lower body muscles (TA, GM, SOL, VL, FDI), the aim of the present study is to explore the neural‐based factors contributing to the observed neuromuscular sex‐related differences by using HDsEMG on biceps brachii (BB), identifying a large motor unit number (Holobar & Farina, 2014; Holobar et al., 2014) at low and high contraction intensities (35–70%MVF), thus analysing lower‐ and higher‐threshold motor units (Martinez‐Valdes et al., 2020). By doing so, we aspire to provide valuable insights into the broad field of neuromuscular physiology, with implications for optimising training protocols, injury prevention strategies and personalised rehabilitation approaches tailored to individual sex‐specific neuromuscular characteristics. A comprehensive understanding of these distinctions may enhance health promotion, well‐being, and performance across diverse populations, thereby contributing novel information to the current scientific literature. Based on previous results (Ahamed et al., 2015; Guo, Jones, et al., 2022; Handelsman et al., 2018; Harrison et al., 2023; Jenz et al., 2023; Lulic‐Kuryllo & Inglis, 2022; Mcdougall et al., 2023; Olmos et al., 2023), potential differences concerning discharge and recruitment properties are expected. Considering existing disparities in hormonal influence on musculoskeletal mechanical properties (Piasecki et al., 2023, 2024; Smith & Woolley, 2004; Velders & Diel, 2013; Wierman, 2007) and previous findings concerning sex‐related differences in motor unit behaviour (Guo, Jones, et al., 2022; Jenz et al., 2023; Nishikawa et al., 2024), higher discharge rate in females and higher recruitment threshold in males are expected.

## 2 METHODS

### 2.1 Ethical approval and participants

The study was approved by the local ethical committee of the University of ‘Foro Italico’, Rome (approval n. CAR157/2023) and conformed to the *Declaration of Helsinki* standards. Twenty‐four recreationally active participants (females, *n* = 13, body mass index (BMI) = 20.2 kg/m^2^, age = 24.3 years; males, *n* = 11, BMI = 25.2 kg/m^2^, age = 24.4 years; mean ± SD) took part in the study; they were assigned unique alphanumeric codes to ensure their privacy and confidentiality. Before enrolling, volunteers were asked to sign a written informed consent document that explained the experimental procedures, outlined potential side effects and assured them of their freedom to withdraw at any time without any consequences. Participants with metabolic disease, upper limb musculoskeletal disorders, acute infection, uncontrolled hypertension, those under medications that impact muscle protein metabolism, modulate vascular tone and neural activity, and those using contraceptives (Burrows & Peters, 2007; Elliott‐Sale et al., 2020; Reif et al., 2021) were excluded. The inclusion criteria comprised an age between 18 and 35 years and good health. Participants’ anthropometric characteristics are reported in Table 1.

**TABLE 1:** Anthropometric, MVF and SST results.

	Group		
Variables	Male	Female	*P*
Age (years)	24.4 ± 1.4	24.3 ± 2.1	0.18
Height (cm)	178.3 ± 5.2	163.7 ± 5.9	**<0.0001**
Body mass (kg)	80.1 ± 11.1	54.0 ± 5.3	**<0.0001**
MVF (N)	372.2 ± 72.6	196.0 ± 30.8	**<0.0001**
BMI (kg/m^2^)	25.2 ± 3.0	20.1 ± 2.0	**<0.0001**
SST (mm)	5.0 ± 2.1	4.5 ± 1.7	0.477

Data are reported as means ± SD. Between‐group comparisons were done with independent sample *t*‐tests. Statistically significant *P*‐values are reported in bold. Abbreviations: BMI, body mass index; MVF, maximal voluntary force; SST, subcutaneous skinfold thickness.

### 2.2 Overview of the study

**FIGURE 1:**
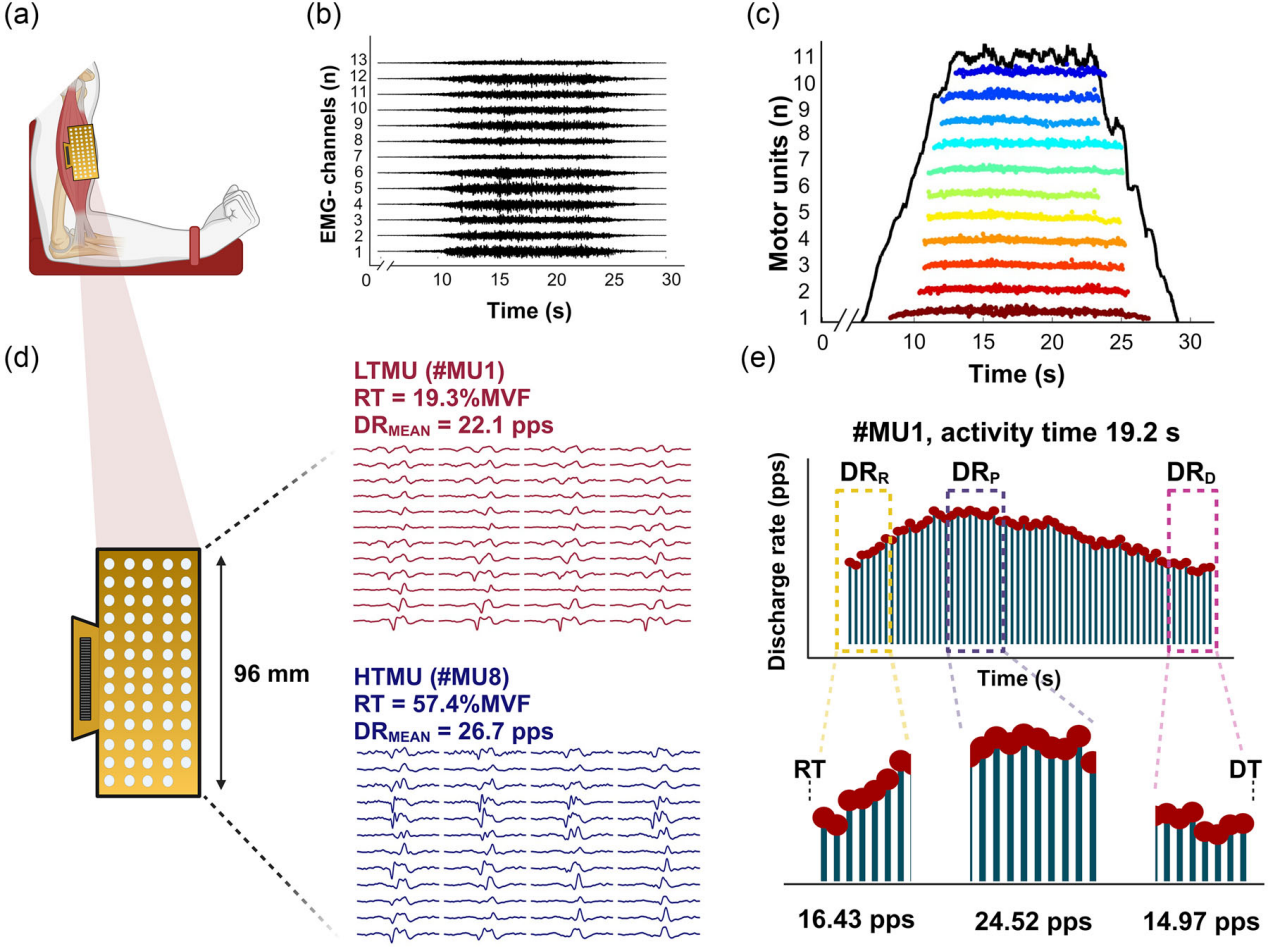
Data acquisition from HDsEMG. (a) A HDsEMG grid, long‐axis oriented and placed over the biceps brachii muscle belly. (b, c) The raw signals (b) were filtered, and the single MU action potentials (c) were obtained after the data were processed in the BSS algorithm. Each coloured stripe reflects a motor unit firing activity associated with the force signal (marked as a full black line). (d) Motor units n#1 and n#8 are displayed as examples of the HDsEMG multichannel signals for a lower‐ and higher‐threshold motor unit from the same recording. RT and DT are calculated as the force values at which motor units are activated and deactivated, corresponding to the first and last spike. (e) Estimation of the discharge rate at recruitment (DR_R_), plateau (DR_P_) and derecruitment (DR_D_). DR_MEAN_, mean discharge rate; DT, derecruitment threshold; HTMU, higher‐threshold motor unit; LTMU, lower‐threshold motor unit; RT, recruitment threshold. Created with BioRender.com.

Participants were asked to be available for two laboratory visits, the first for familiarisation and the second for the neuromuscular tests and data acquisition, separated by 48–72 h. During the first visit, volunteers received the experimental and testing procedure information and were familiarised with maximal voluntary isometric contractions (MVICs) and trapezoidal isometric contractions (ramp) of their elbow flexors, self‐reporting the upper limb dominance, while no measurements were made. Data acquisition was performed during the second visit, including the recordings of elbow flexion MVF, submaximal contraction forces, and HDsEMG recordings from the biceps brachii muscle (Figure 1a). In addition, subcutaneous skinfold thickness (SST) was assessed on biceps brachii using plicometry (Mei et al., 2002). According to previous findings, female participants performed the test during either the ovulatory or the mid‐luteal phase to reduce neuromuscular activity fluctuations and to avoid a documented general‐activation decrease attributed to the early follicular phase (Tenan et al., 2013; Weidauer et al., 2020).

### 2.3 Experimental procedures

Volunteers were asked to refrain from strenuous exercise and caffeine consumption 48 and 24 h before the tests. The testing procedures were performed after a standardised warm‐up, consisting of 3 × 30 isometric BB contractions at a low intensity of the perceived MVF (30–40%MVF) separated by 30 s. They were asked to focus on the movement of the elbow flexors and isolate the BB intervention as much as possible during the warm‐up and testing procedures. Consequently, the participants performed three MVICs with 180 s of rest between trials, and they were asked to push as hard as possible for 5 s, receiving a strong verbal incitation. The MVF was set as the highest value recorded across the three MVICs to obtain the submaximal contraction target forces. After 5 min from the last maximal trial, participants performed trapezoidal contractions in a randomised order separated by 5 min. The ramps consisted in a linear force increase at a rate of 5%MVF per second to a target value (35% or 70%MVF), which was maintained for 10 s, then, a linear force decrease back to the resting value at the same rate as the increasing phase.

### 2.4 Force signal recording

The elbow flexion force of the dominant limb was assessed with an isokinetic dynamometer (Kin‐Com, Chattanooga, TN, USA). Participants were seated comfortably in the dynamometric chair and were stabilised by chest and waist straps. The position of the upper arm was parallel to the trunk, with the forearm halfway between pronation and supination and an elbow flexion of 90°. The centre of rotation of the lever arm was aligned with the distal lateral epicondyle of the humerus, and the wrist was secured in a cuff attached to the load cell (Kin‐Com), which consisted of four strain gauges. The analog force signal was amplified and sampled at 2048 Hz with an external analog‐to‐digital (A/D) converter (EMG‐400, OT‐Bioelettronica, Turin, Italy) and synchronised with the electromyogram. Since the trapezoidal contractions needed to be visually guided, a trapezoidal pattern was shown to participants during the contraction, with a minimum/maximum error of 3% MVF.

### 2.5 High‐density surface electromyography recordings

HDsEMG signals were recorded from the BB with an adhesive grid of 64 electrodes (13 rows × 5 columns; gold‐coated; diameter: 1 mm; inter‐electrode distance (IED): 8 mm; OT‐Bioelettronica, Turin, Italy) (Figure 1d). After skin preparation (shaving, light skin abrasion and 70% ethanol cleansing), the muscle perimeter was identified through palpation and marked by a surgical pen. The grid orientation was based on recordings from a 16‐electrodes array (IED 5 mm, OT‐Bioelettronica), identifying the innervation zone (IZ) to estimate the fibre direction, as described in Lecce et al. (2023a). The IZ was located by identifying the inversion point in the action potential propagation direction proximally (towards BB proximal tendon) and distally (towards BB distal tendons) along the electrode column. The grid was placed right over the IZ (Huang et al., 2019) over the muscle belly, using a disposable biadhesive with layer holes adapted to the HDsEMG grids (SpesMedica, Battipaglia, Italy). The foam layer holes were filled with a conductive paste (SpesMedica) to ensure skin‐electrode contact. Reference electrodes were positioned on the styloid process of the ulna, on the acromion skin surface and on the medial malleolus. HDsEMG signals were recorded in monopolar mode and converted to digital data by a 16‐bit multichannel amplifier (EMG‐Quattrocento, OT Bioelettronica), amplified (×150), sampled at 2048 Hz and band‐pass filtered (10−500 Hz) before being stored for offline analysis.

### 2.6 Force and HDsEMG analysis

The force signal was converted to newtons, and gravity compensation removed the offset. The signal was low‐pass filtered with a fourth‐order, zero‐lag Butterworth filter with a cut‐off frequency of 15 Hz. Only trapezoidal contractions without any counter‐movement action or pre‐tension were analysed (Lecce et al., 2023b). Before decomposing into individual motor unit action potentials, the monopolar EMG recordings were band‐pass filtered at 20−500 Hz (second‐order, Butterworth). The raw HDsEMG signals (Figure 1b) were decomposed using the convolutive blind source separation algorithm (BSS) (Holobar & Zazula, 2007). This decomposition procedure can identify motor unit discharge times (Figure 1c) over broad force ranges (Holobar & Farina, 2014) by multiple comparisons of the recorded action potential waveforms.

An experienced investigator manually analysed all the identified motor units and retained only those characterised by a high pulse‐to‐noise (>30 dB) (Holobar et al., 2014). The recruitment and derecruitment thresholds (RT and DT, respectively) were identified as the absolute (N) and the normalised (%MVF) force values at which MUs were activated and deactivated, identifying the first and the last spikes, respectively (Del Vecchio, Casolo, et al., 2019). The total number of discharges in the cumulative spike train was divided by the number of active motor units across the bouts, providing the average number of discharges per motor unit per second (DR_MEAN_). This value was related to the average neural drive to motoneurons (Del Vecchio, Negro, et al., 2019, 2018) across the whole ramp.

The average discharge rate for each MU was assessed during the recruitment (DR_R_), plateau (DR_P_) and derecruitment (DR_D_) phases. The fluctuation of the firing rate was assessed with the interspike interval variability (ISIv), the variability of consecutive motor unit spikes, during the plateau phase of the trapezoidal ramps. According to previous studies, only those motor units showing ISIv values below 40% of fluctuation were considered (Lee & Heckman, 1999; Tracy et al., 2005). The DT–RT, DR_P_–RT and ISIv–RT relationships were calculated for each participant. Moreover, these motor unit values were classified as a function of their recruitment thresholds; by doing so, motor unit properties were normalised and can be compared in two different populations (males and females).

Since motor unit characteristics include the onion skin phenomenon, in which earlier recruited MUs generally show a higher discharge rate than those of later recruited (De Luca & Hostage, 2010), and a phenomenon called reverse‐onion skin, in which later recruited motor units have higher discharge rate than those of earlier MUs (Inglis & Gabriel, 2021a), the whole motor unit pool was classified as lower‐threshold (LTMUs) and higher‐threshold (HTMUs) in order to compare absolute and relative results considering differences of different motor unit populations (Nishikawa et al., 2024) by including in LTMUs all those characterised by recruitment thresholds ≤30%MVF and in HTMUs all those having recruitment thresholds >30%MVF (Valli et al., 2023); cut‐off has been arbitrarily chosen based on the data distribution as previously described (Martinez‐Valdes et al., 2020; Nishikawa et al., 2024).

The input–output gain of motor neurons was additionally estimated by computing the change in discharge rate during the plateau phase relative to that at recruitment (∆DR) as a function of the change in relative force from recruitment to the plateau phase (∆F) as linear regression. This analysis reflects an estimate of the input–output gain of the motor neuron pool since it reflects the extent of change in generated force as a response to the change in the firing rate needed to the relative exertion (Del Vecchio, Casolo, et al., 2019; Ferguson & Cardin, 2020; Petrovic et al., 2023), thus providing information through the estimation of the final step to all motor command (Binder et al., 2020).

### 2.7 Statistical analysis

The Shapiro–Wilk test assessed the data distribution normality. Multiple independent Student's *t*‐tests were used to compare sex‐related differences in the average number of identified motor units, SST and anthropometrical characteristics. Multi‐level mixed‐effect linear regression analysis was used to compare sex differences in the DR_MEAN_, DR_R_, DR_P_, DR_D_, ISIv, the absolute and normalised RT and DT, DT/RT, DR/RT and the ISIv/RT to better incorporate the whole sample of extracted MUs (not just the mean values obtained from each participant), which preserves variability within and across participants simultaneously to the greatest extent, as described in previous studies investigating motor unit parameters (Guo, Jones, et al., 2022). The input–output gain of motoneurons and the DT/RT, DR/RT and ISIv/RT ratios were assessed by comparing the slopes with a *t*‐test; this analysis was performed considering each participant's slope. Cohen's *d* was calculated to assess the effect size when the *t*‐test result was statistically significant. The partial eta squared was used to evaluate the effect size of mixed‐effect model analysis results. The correlations were analysed through Pearson's correlation coefficient test. Comparisons between slopes were performed with univariate linear regression tests. The statistical calculations were performed using SPSS Statistics 25.0 (IBM Corp., Armonk, NY, USA) and jamovi 2.3.18 (The jamovi project, Sydney, Australia). *P *< 0.05 was considered a statistically significant result. Data are reported as the means ± SD in the text.

## 3 RESULTS

Anthropometric, MVF and SST results are reported in Table 1, and myoelectrical differences are summarised in Table 2.

**TABLE 2:** Motor unit comparisons.

	Low‐threshold motor units			High‐threshold motor units		
	Male	Female	*P*	Male	Female	*P*
DR_MEAN_ (pps)	17.0 ± 2.0	21.2 ± 2.4	**<0.0001**	21.8 ± 2.6	19.4 ± 2.9	**0.04**
DR_R_ (pps)	12.5 ± 1.62	14.8 ± 1.9	**0.006**	15.5 ± 2.1	13.0 ± 1.7	**0.005**
DR_P_ (pps)	17.7 ± 2.4	21.9 ± 2.8	**0.001**	23.2 ± 2.9	20.6 ± 2.9	**0.04**
DR_D_ (pps)	10.4 ± 0.5	12.3 ± 1.6	**0.001**	13.7 ± 1.4	11.9 ± 1.7	**0.01**
ISIv (%)	21.4 ± 4.2	23.2 ± 2.9	0.21	25.9 ± 5+3.3	28.8 ± 2.7	**0.03**
RT (N)	83.7 ± 17.7	38.3 ± 4.7	**<0.0001**	178.0 ± 22.6	84.0 ± 10.5	**<0.0001**
DT (N)	73.6 ± 17.4	32.2 ± 6.2	**<0.0001**	173.5 ± 18.3	80.4 ± 14.1	**<0.0001**
RT (%MVF)	22.2 ± 2.4	19.5 ± 2.5	**0.004**	47.8 ± 3.4	42.8 ± 4.4	**0.001**
DT (%MVF)	19.6 ± 3.7	16.4 ± 2.7	**0.01**	46.6 ± 4.1	40.9 ± 5.4	**0.001**
DT/RT	0.88 ± 0.12	0.86 ± 0.07	0.712	0.98 ± 0.09	0.94 ± 0.06	0.277
DR/RT	0.85 ± 0.24	1.21 ± 0.31	**0.004**	0.49 ± 0.06	0.5 ± 0.10	0.919
ISIv/RT	0.97 ± 0.19	1.25 ± 0.2	**0.002**	0.54 ± 0.07	0.68 ± 0.09	**0.001**

Data are reported as means ± SD. Statistically significant *P*‐values are reported in bold. DR, discharge rate; DT, derecruitment threshold; ISIv, interspike interval variability; RT, recruitment threshold.

### 3.1 Motor unit identification

**FIGURE 2:**
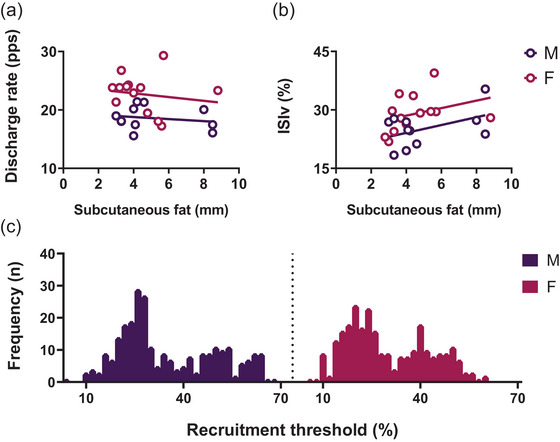
The relationship between subcutaneous fat, myoelectrical parameters and identified motor unit distribution. (a, b) The average discharge rate (DR) and the interspike variability (ISIv) for each participant, as determined through raw data analysis, are presented in relationship to subcutaneous fat measurements obtained through the assessment of SST in (a) and (b), respectively. (c) The distribution of identified motor units by their recruitment threshold of male and female participants.

A total of 540 motor units were identified; 271 were found in males and 269 in females (Figure 2c). The average number of identified motor units per male subject in each trial was 24.6 ± 2.7, whereas per female subject it was 20.6 ± 2.5 (*P* = 0.001, *d* = 1.53). Considering the LTMUs, an average of 13.4 ± 2.9 and 11.7 ± 1.84 motor units were identified in males and females, respectively (*P* = 0.04, *d* = 0.26). For the HTMUs, an average of 11.2 ± 2.1 and 9.0 ± 1.2 were identified in males and females, respectively (*P* = 0.005, *d* = 1.28).

### 3.2 Subcutaneous skinfold thickness and MVF

Male and female participants exhibited similar values of subcutaneous fat, with no statistically significant differences observed between the two groups (*P* > 0.05). The correlations between the average DR and SST were found to be non‐significant among male participants (*R* ^2^ = 0.03, *P* > 0.05; see Figure 2a) as well as among female participants (*R* ^2^ = 0.02, *P* > 0.05; see Figure 2b), underlining that there is no between‐sex difference in the influence of subcutaneous fat on the MU DR. No statistically significant correlations emerged between the ISIv and SST in either male (*R* ^2^ = 0.21, *P *> 0.05) or female (*R* ^2^ = 0.10, *P *> 0.05) participants. When comparing the slopes, no statistically significant differences were observed between male and female groups in the DR–SST and ISIv–SST relationship (*P *> 0.05). Male participants exhibited significantly higher MVF than females (*P *< 0.0001, *d* = 3.15).

### 3.3 Discharge properties

**FIGURE 3:**
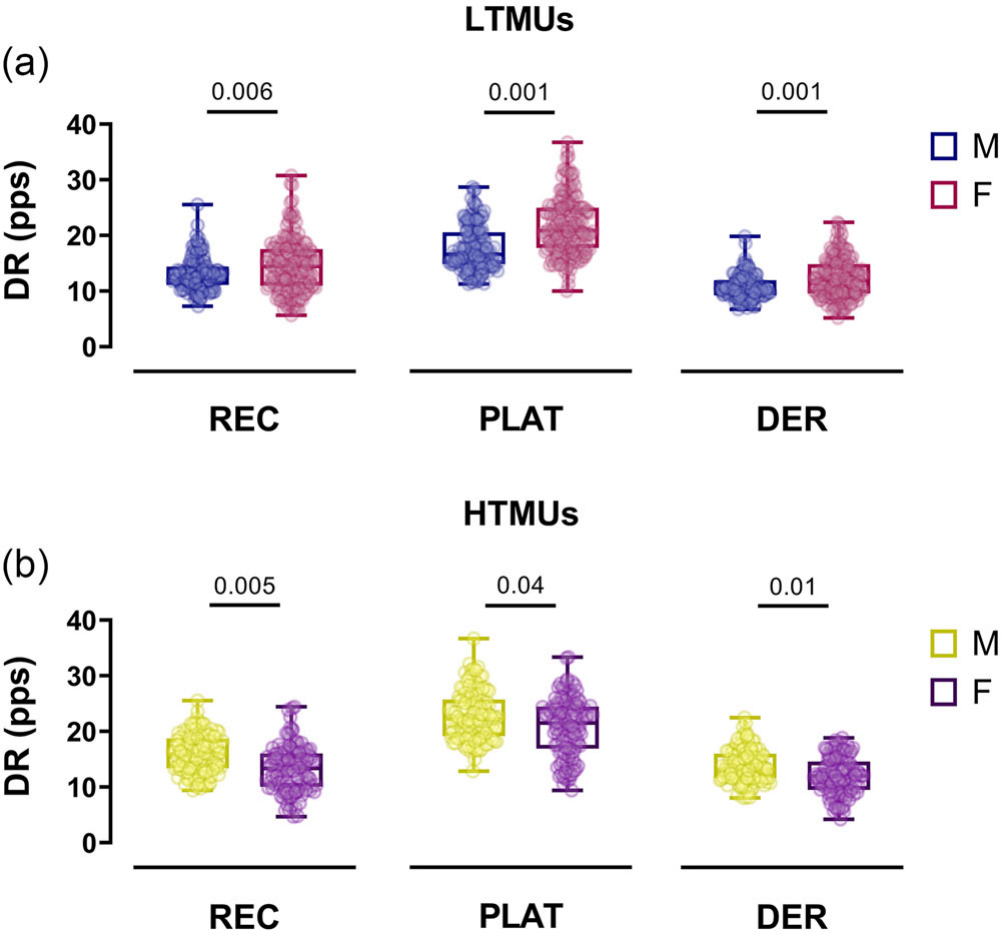
Discharge rate comparisons. Swarm plots of male‐female comparisons concerning the discharge rate at recruitment (REC), plateau (PLAT), and derecruitment (DER) are displayed for LTMUs (a) and HTMUs (b). Each circle represents a single participant. Data are reported as the mean ± SD. DER, derecruitment; DR, discharge rate; HTMUs, higher‐threshold motor units; LTMUs, lower‐threshold motor units; PLAT, plateau; REC, recruitment.

In LTMUs, the DR_MEAN_ exhibited a significantly higher value in the female group when compared to the male group (*P *< 0.0001, η_p_^2^ = 0.664). This result was accompanied by higher values for DR_R_ (*P* = 0.006, η_p_^2^ = 0.531), DR_P_ (*P* = 0.001, η_p_^2^ = 0.563) and DR_D_ (*P* = 0.001, η_p_^2^ = 0.612) in females, with greater values than males (Figure 3a). No significant differences were observed in the ISIv (*P* = 0.211). In HTMUs, males exhibited a significantly higher DR_MEAN_ than females (*P* = 0.04, *d* = 0.88). Additionally, sex‐related disparities were observed in DR_R_ (*P* = 0.005, η_p_^2^ = 0.482), DR_P_ (*P* = 0.04, η_p_^2^ = 0.362) and DR_D_ (*P* = 0.01, η_p_^2^ = 0.397), with higher values in the male group (Figure 3b). The female sample displayed a significantly higher ISIv (*P* = 0.03, η_p_^2^ = 0.132).

**FIGURE 4:**
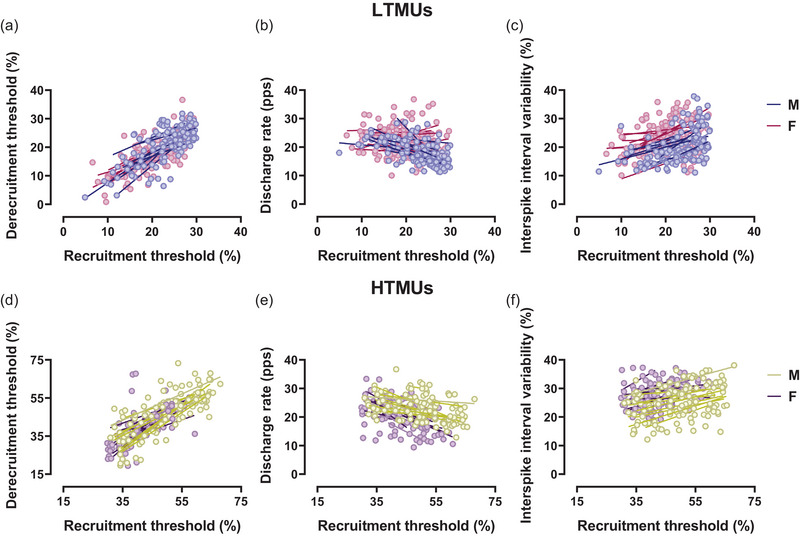
Sex‐related differences in DT, DR and ISIv as a function of the recruitment threshold. (a–c) Scatter plots of lower‐threshold motor unit results for DT–RT (a), DR–RT (b) and ISIv–RT (c). (d–f) Higher‐threshold motor unit results for DT–RT (d), DR–RT (e) and ISIv–RT (f). Each circle represents an identified motor unit. Single slopes per participant are reported for both male and female participants. DT, derecruitment threshold; ISIv, interspike interval variability; RT, recruitment threshold.

**FIGURE 5:**
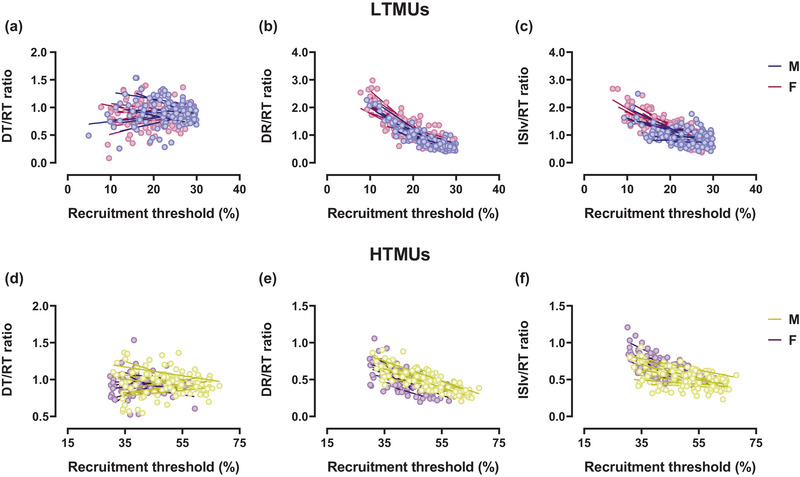
Sex‐related differences in the DT/RT, DR/RT and ISIv/RT ratios as a function of the recruitment threshold. (a–c) Scatter plots of lower‐threshold motor unit results for DT/RT (a), DR/RT (b) and ISIv/RT (c) ratios. (d–f) Higher‐threshold motor unit results for DT/RT (d), DR/RT (e) and ISIv/RT (f) ratios. Each circle represents an identified motor unit. Single slopes per participant are reported for both male and female participants. DT, derecruitment threshold; ISIv, interspike interval variability; RT, recruitment threshold.

Correlation analyses revealed a significant inverse relationship between DR_P_ and RT in LTMUs for both males (*R* ^2^ = 0.30, *P *< 0.0001) and females (*R* ^2^ = 0.04, *P* = 0.04), displayed in Figure 4b. The present result was confirmed by the DR/RT ratio to RT correlation, which is significant in males (*R* ^2^ = 0.79, *P *< 0.0001) and females (*R* ^2^ = 0.64, *P *< 0.0001), as displayed in Figure 5b. Additionally, a significant correlation was observed in the analysis between ISIv and RT in the male group (*R* ^2^ = 0.05, *P* = 0.003), whereas no significant results were found in the female group (*R* ^2^ = 0.006, *P *> 0.05), displayed in Figure 4c. The ISIv/RT ratio to RT correlation was significant in both the male (*R* ^2^ = 0.33, *P *< 0.0001) and female groups (*R* ^2^ = 0.49, *P *< 0.001), displayed in Figure 5c. In HTMUs, a significant correlation emerged between DR_P_ and RT in males (*R* ^2^ = 0.08, *P* = 0.001), while no significant correlation was observed in females (*R* ^2^ = 0.01, *P *> 0.05), displayed in Figure 4e. However, the male (*R* ^2^ = 0.66, *P *< 0.0001) and the female (*R* ^2^ = 0.36, *P *< 0.0001) groups showed significant results concerning DR/RT ratio to recruitment, as shown in Figure 5e. In addition, the ISIv/RT correlation was significant in males (*R* ^2^ = 0.11, *P* = 0.002) but not in females (*R* ^2^ = 0.01, *P *> 0.05), as displayed in Figure 4f. Nevertheless, the correlation between the ISIv/RT ratio and RT was statistically significant in both the male (*R* ^2^ = 0.24, *P *< 0.0001) and female groups (*R* ^2^ = 0.32, *P *< 0.0001), as displayed in Figure 5f.

### 3.4 Recruitment properties

**FIGURE 6:**
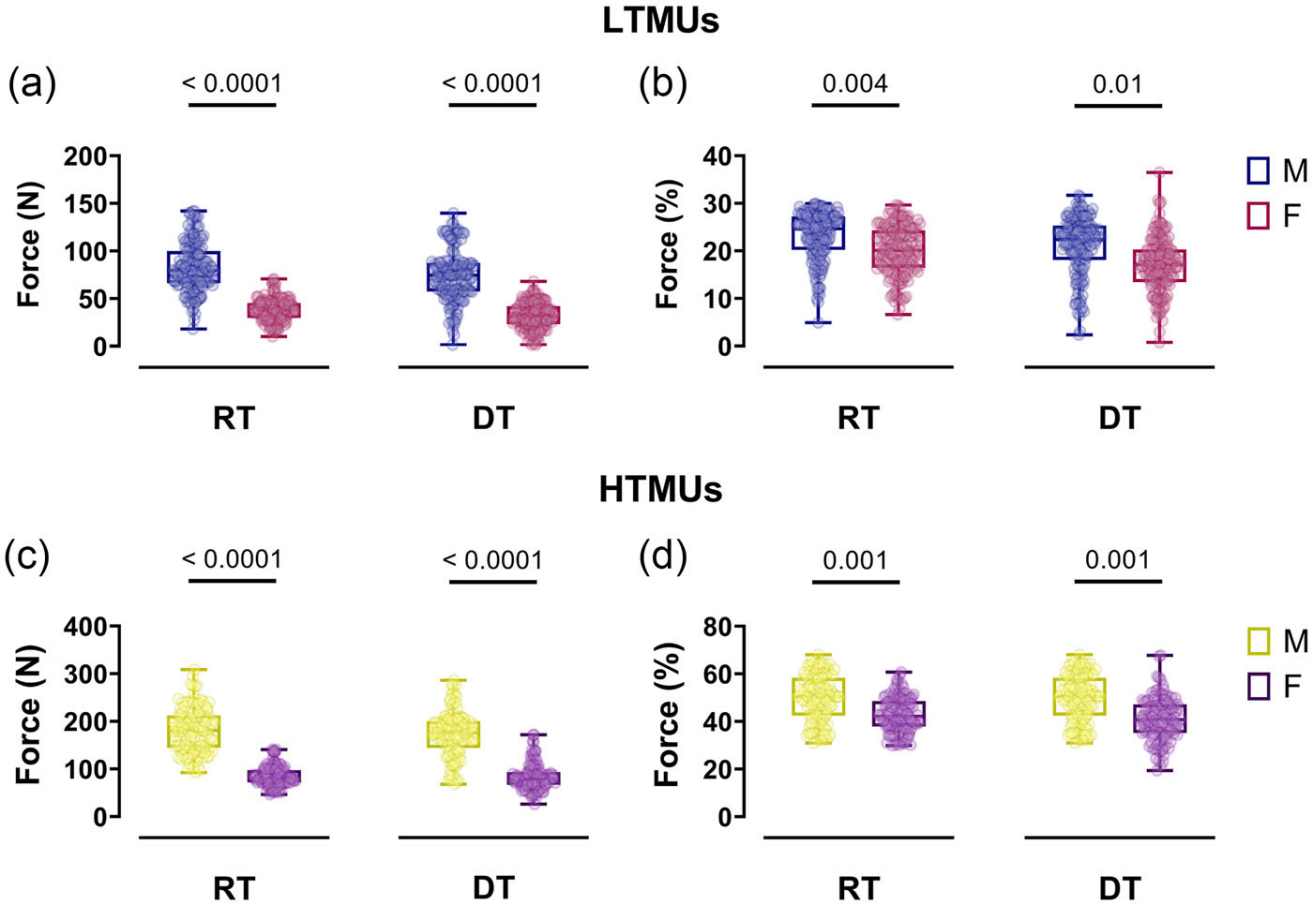
Recruitment and derecruitment threshold comparisons. (a,c) Swarm plots of male‐female comparisons in the absolute recruitment and derecruitment threshold in LTMUs (a) and HTMUs (c). (b,d) The relative recruitment and derecruitment threshold for LTMUs (b) and HTMUs (d). Data are reported as the mean ± SD. Each circle represent a single participant. DT,derecruitment threshold; HTMUs,higher‐threshold motor units; LTMUs, lower‐threshold motor units; MVF, maximal voluntary force; RT, recruitment threshold.

The absolute RT and DT exhibited significantly higher values in the male group (*P *< 0.001) observed in lower‐ (RT: η_p_^2^ = 0.713; DT: η_p_^2^ = 0.724) and higher‐threshold motor units (RT: η_p_^2^ = 0.853; DT: η_p_^2^ = 0.872) as shown in Figure 6a–c. Similarly, when normalising as a %MVF, the RT and DT remained significantly higher in the male group across all levels of contraction intensities (LTMUs: RT: *P* = 0.004, η_p_^2^ = 0.384; DT: *P* = 0.01, η_p_^2^ = 0.311; HTMUs: RT: *P* = 0.001, η_p_^2^ = 0.402; DT: *P* = 0.001, η_p_^2^ = 0.412), as displayed in Figure 6b–d.

Significant DT/RT relationships were found in both the male (*R* ^2^ = 0.49, *P *< 0.001) and female (*R* ^2^ = 0.48, *P *< 0.001) lower‐threshold motor units, as displayed in Figure 4a. Furthermore, the DT/RT ratio to RT relationships revealed non‐significant correlations in both males (*R* ^2^ = 0.06, *P *> 0.05) and females (*R* ^2^ = 0.004, *P *> 0.05), underscoring the similarity in RT values to DT of both groups (Figure 5a). Within HTMUs, significant correlations in the DT/RT relationship were identified for both the male (*R* ^2^ = 0.50, *P *< 0.001) and female groups (*R* ^2^ = 0.49, *P *< 0.001), as displayed in Figure 4d. These findings were reflected in the DT/RT ratio to RT correlations, which were non‐significant in both males (*R* ^2^ = 0.03, *P *> 0.05) and females (*R* ^2^ = 0.003, *P *> 0.05), as shown in Figure 5d.

### 3.5 Firing and recruitment patterns

**FIGURE 7:**
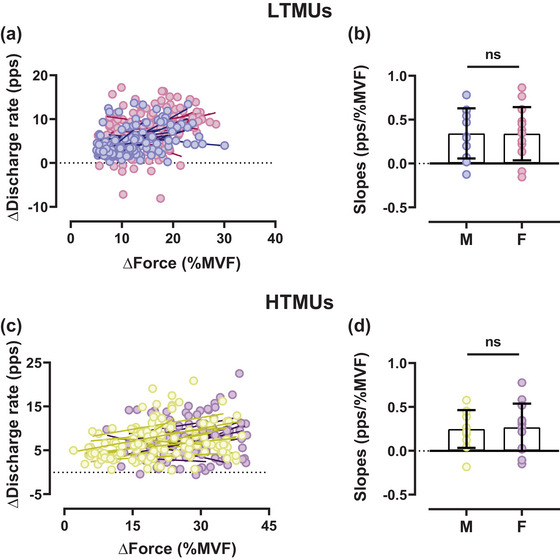
Input–output slope comparisons of male and female motor units. (a, c) Scatter plots of LTMUs (a) and HTMUs (c) representing the differences in force and discharge rate between the plateau and the recruitment phases during the ramp contraction, reflecting the input–output gain of motoneurons. Each circle represents an identified motor unit. (b, d) The slope differences for LTMUs (b) and HTMUs (d) displayed as aligned dot plots. Data are reported as the mean ± SD. HTMUs, higher‐threshold motor units; LTMUs, lower‐threshold motor units.

Sex‐related differences were observed in the slope comparison between the DR and the recruitment threshold in LTMUs (*P* = 0.003, η_p_^2^ = 0.234), while non‐significant differences were observed in HTMUs. However, non‐significant results were evidenced in the DR/RT ratio to RT relationships for lower‐ and higher‐threshold motor units (*P *> 0.05). Non‐significant differences were also observed by the slope comparisons of the ISIv to RT relationship for both LTMUs and HTMUs (*P *> 0.05). However, significant slope differences were found in the ISIv/RT ratio to RT for both lower‐threshold (*P* = 0.02, η_p_^2^ = 0.112) and higher‐threshold (*P* = 0.001, η_p_^2^ = 0.231) motor units. There were no significant differences when comparing the DT to RT relationship across all intensities (*P *> 0.05). Furthermore, non‐significant results were obtained for the DT/RT ratio to RT comparisons in both lower‐ and higher‐threshold motor units (*P *> 0.05). No significant differences were observed in the input–output gain of motor neurons in either lower‐threshold (*P* = 0.256) or higher‐threshold (*P* = 0.143) motor units, as displayed in Figure 7.

## 4 DISCUSSION

In the present study, we explored sex‐related differences in a relatively large sample of motor units using high‐density surface electromyography. This investigation helepd identify distinct neural control strategies in indivudals of different sexes, offering deeper insights into discharge and recruitment behaviour of lower‐ and higher‐threshold motor units. Fewer motor units per participant were identified in the female group compared to the male one, supporting previous evidence reporting higher number of units found in males (Taylor et al., 2022). This difference does not seem to be associated with subcutaneous fat, which was similar between males and females in the present study. In addition, we observed a higher maximal voluntary force in male individuals. The documented strength disparities have been previously associated with various determinants, such as diversities in peripheral neuromuscular characteristics, sex hormone impact, fibre size and body composition (Haizlip et al., 2015; Inglis & Gabriel, 2020; Landen et al., 2023; Piasecki et al., 2021; Taylor et al., 2022), suggesting possible influences on motor unit behaviour. Indeed, significant differences emerged in the discharge patterns of lower‐ and higher‐threshold motor units analysed in ramp contractions. Specifically, we observed a higher rate coding in lower‐threshold motor units of females, supporting previous research (Guo, Jones, et al., 2022; Jenz et al., 2023). In contrast, a converse pattern emerged in male HTMUs, displaying greater discharge rate. This study details the differences in firing rates between male and female individuals during recruitment, plateau and derecruitment stages, thereby providing a characterisation of sex‐based distinctions in lower‐ and higher‐threshold motor unit discharge strategies not only for initial and sustained phases (Inglis & Gabriel, 2020; Lulic‐Kuryllo & Inglis, 2022) but also for derecruitment. Nevertheless, a significant sex‐based difference was observed in the association between the discharge rate and recruitment threshold only in lower‐threshold motor units, indicating a differential synaptic input to motor neurons for that population. Since we observed differences in the discharge properties even when normalised by the recruitment threshold (DR/RT ratio), it is plausible that males and females display dissimilar rate coding at similar motor unit sizes. This ratio also serves as a reliable index for normalising firing rates with respect to motor unit recruitment thresholds, taking into account the inherent differences in firing strategies between small and large motor units across varying force levels (De Luca & Hostage, 2010).

In addition, it has been reported that higher discharge rates are associated with lower muscle mass involved in a given exertion (Duchateau et al., 2006). Since it is established that female muscle mass is significantly less than that of males, along with a different percentages of fibre composition (Haizlip et al., 2015), it is plausible that lower‐threshold motor units need a higher firing rate to sustain force exertion at specific %MVF in females, whereas higher‐threshold MUs do not. Since previous results accounted for matched MU discharge rates in the 60–80%MVF range (Parra et al., 2020), the observed differences in the absolute firing rate may depend on the participants' characteristics. Additionally, when comparing the DR/RT ratio, a notable inverse correlation is evident in female and male LTMUs, consistent with prior research (Jesunathadas et al., 2010). These results were accompanied by a higher neural drive observed in female LTMUs and a greater DR/RT ratio and firing rates across all ramp phases, underlining not only higher rate coding across the whole task (Guo, Jones, et al., 2022; Jenz et al., 2023; Piasecki et al., 2024) but also during distinct phases, represented by recruitment, plateau and derecruitment. These distinctions may be attributable to variations in common input associated with descending neural drive and mechanical output, implying greater firing activity in male motor units (Del Vecchio, Falla, et al., 2019; Farina et al., 2014). However, these hypotheses concerning sex‐dependent differences warrant further exploration, particularly during sustained contractions (De Luca & Erim, 1994; Farina et al., 2014). Prior research demonstrated that muscle fibre diameter is positively associated with the motor unit recruitment thresholds (Casolo et al., 2023; Del Vecchio, Negro, et al., 2018). According to these findings, the present study underscores that female motor units manifest distinct firing strategies and similar neural drive considering motor unit size during low‐intensity contractions, a pattern that inversely aligns with the findings at higher intensities, suggesting a greater neural drive among male HTMUs in biceps brachii. As reported previously (Olmos et al., 2023), the differences in fibre composition may affect motor unit properties since myosin heavy chain expression in VL muscle is different comparing males and females (Haizlip et al., 2015).

In contrast to previous investigations (Lulic‐Kuryllo & Inglis, 2022; Piasecki et al., 2021), the current study offers a deeper insight into sex‐related differences in firing strategies by analysing individual motor unit properties relative to their recruitment threshold. Our results also indicated significant disparities in the interspike interval variability (ISIv), with females displaying broader fluctuations for higher‐threshold units even when normalised by recruitment threshold (ISIv/RT ratio). This observation supports prior research indicating more discharge variability in female individuals (Inglis & Gabriel, 2021b) and suggests that these differences may also be reflected in the force steadiness analysed with the coefficient of variation of force (Dideriksen et al., 2012; Tracy et al., 2005). These results provided novel insights into the discharge rate fluctuation, likely supported by the positive association between testosterone and the variability in motor unit firing rates. Indeed, the observed distinctions have been previously attributed to the different testosterone levels, as it has been demonstrated to positively affect the neuromuscular system by decreasing firing variability and reducing potentials dispersion along axonal branches and muscle fibres (Guo, Piasecki, et al., 2022). This likely explains the sex‐related differences in the ISIv and the firing rate, especially in higher‐threshold units, bringing novel information concerning male and female motor unit characterisation and behaviour.

According to our results, male and female individuals also displayed differential recruitment properties, with higher recruitment and derecruitment thresholds in male motor units. However, the association between recruitment and derecruitment thresholds is comparable between sexes in both LTMU and HTMU populations, reflecting no differences in the activation and deactivation patterns. Indeed, the disparities found in the absolute and normalised recruitment and derecruitment thresholds likely stem from dissimilar muscle fibre composition (Landen et al., 2023; Olmos et al., 2023), as well as the potential influence of sex hormones on the central nervous system (Haizlip et al., 2015; Handelsman et al., 2018; Piasecki et al., 2024; Smith & Woolley, 2004; Wierman, 2007). Nevertheless, the higher recruitment and derecruitment thresholds found in males may depend on a larger number of higher‐threshold units identified by the BSS algorithm, considering possible influences of the abovementioned aspects. The association between the force exerted and the discharge rate during the ramp pahse of contractions (plateau *minus* recruitment), reflecting the input–output gain of motor neurons, underlined similarities in the response amplitude to a certain neural input (Devanne et al., 1997). Hence, no differences in the mechanical response to input from the corticomedullary tract were hypothesised, suggesting similar modulation of spinal motor neuron activity by the motor cortex for both sexes (Binder et al., 2020; Del Vecchio, Casolo, et al., 2019; Nuzzo et al., 2017).

In summary, we characterised significant sex‐related differences in motor unit discharge strategies from biceps brachii, which is not typically taken into account for sex‐difference comparisons, referring to distinct patterns in rate coding (lower‐ and higher‐threshold MUs) and DR/RT ratios (lower‐threshold MUs). The higher ISIv observed in females also underlined potential influences of structural and hormonal diversities. The greater RT and DT found in male motor units have been attributed to differing muscle fibre compositions. Importantly, our findings revealed no significant sex‐related differences in the DT/RT ratio, indicating similar motor unit activation–deactivation strategies. Our study provides novel insights by analysing higher‐threshold motor units and employing normalised comparisons within a broad motor unit pool. This comparison of relatively large sample of motor units in the upper limbs between sexes suggests that differences previously attributed to muscle factors may be instead sex‐dependent. Acknowledging that multifaceted physiological parameters underpin these sex‐related differences in motor unit behaviour is imperative. While considerable strides have been made, technological advancements continue to expand our understanding of neuromuscular physiology. Future research prospects hinge on the potential for further elucidating the intricacies beneath the surface of this complex physiological characterisation.

### 4.1 Limitations

In the present study, we characterised a relatively large number of motor units in male and female participants by applying HDsEMG. However, the optimal use of this approach involves isometric conditions, which may limit insights into dynamic tasks. Furthermore, given the broad range of identified motor unit populations, we classified LTMUs and HTMUs by their recruitment threshold rather than by the contraction intensity at which they were recorded (35−70%MVF) to minimise the higher firing variability of LTMUs identified in higher‐intensity trials.

## AUTHOR CONTRIBUTIONS

All authors participated equally in the conception of the work, acquisition and data analysis. EL and IB drafted the work; all authors revised the final version. All authors have read and approved the final version of this manuscript and agree to be accountable for all aspects of the work in ensuring that questions related to the accuracy or integrity of any part of the work are appropriately investigated and resolved. All persons designated as authors qualify for authorship, and all those who qualify for authorship are listed.

## CONFLICT OF INTEREST

None declared.

## Data Availability

The data supporting the findings of this study are available on request from the corresponding author.
